# Aging influences nucleolar responses to traumatic brain injury in Drosophila

**DOI:** 10.1371/journal.pone.0335715

**Published:** 2025-11-03

**Authors:** Stacey A. Rimkus, Rebeccah J. Katzenberger, Barry Ganetzky, David A. Wassarman

**Affiliations:** 1 Department of Medical Genetics, School of Medicine and Public Health, University of Wisconsin-Madison, Madison, Wisconsin, United States of America; 2 Department of Genetics, College of Agricultural and Life Sciences, University of Wisconsin-Madison, Madison, Wisconsin, United States of America; University of British Columbia, CANADA

## Abstract

Traumatic brain injury (TBI) affects millions of people globally each year, yet effective treatments remain limited. A major challenge is the complexity of cellular and molecular responses to brain injury, many of which overlap with those seen in aging. A key hallmark of aging is nucleolar enlargement in brain and other tissues, reflecting increased ribosome biogenesis. Nucleolar size is regulated by the target of rapamycin (TOR) signaling pathway, which during aging is aberrantly activated. Inhibiting TOR reduces nucleolar size and extends lifespan in several model organisms. Using a *Drosophila melanogaster* model of closed-head TBI, we investigated whether injury influences nucleolar dynamics. Immunofluorescence microscopy of fibrillarin, a major nucleolar protein, revealed that brains of young, injured flies had substantially larger nucleoli than uninjured controls within one day of injury. Over the following weeks, the difference gradually diminished as nucleolar size increased in uninjured flies, eventually matching that of injured flies, which remained relatively stable. Additionally, heterogeneity in nucleolar size across cells became more pronounced with injury and aging. Finally, injury of older flies resulted in little or no nucleolar enlargement and even shrinkage within a few days of injury. These results suggest that TBI and aging converge on shared mechanisms that regulate nucleolar size, which may reach a maximal limit through either process. Consistent with this, mortality at 24 hours post-injury in young flies was significantly reduced by pharmacological inhibition of TOR with rapamycin or RapaLink-1, indicating that nucleolar enlargement contributes to TBI-induced damage. Overall, our results suggest that TBI accelerates the aging-associated increase in nucleolar size, implicating elevated ribosome biogenesis in TBI pathogenesis and highlighting TOR inhibition as a promising therapeutic approach.

## Introduction

Traumatic brain injury (TBI) affects approximately 70 million people worldwide each year [1,2]. However, its underlying mechanisms remain poorly understood due to the complex, evolving cascade of secondary cellular and molecular responses influenced by both genetic and environmental factors [3–5]. Emerging evidence suggests that secondary responses may impair recovery by accelerating normal aging processes [6,7]. Indeed, several hallmarks of aging, such as chronic inflammation, oxidative stress, mitochondrial dysfunction, and neurodegeneration, are exacerbated by TBI [6–11].

Another hallmark of aging is altered nucleolar structure and function, which reflects changes in ribosome biogenesis and cellular stress responses. Nucleoli are specialized nuclear compartments where ribosomal DNA (rDNA) is transcribed, ribosomal RNA (rRNA) is processed, and ribosomal subunits are assembled [12,13]. They are composed of three distinct regions: the fibrillar center (FC), the dense fibrillar component (DFC), and the granular component. (GC) [14]. The DFC contains fibrillarin, a protein essential for rRNA processing. In many cell types, nucleolar size, as indicated by fibrillarin staining, increases with age [15,16]. For example, in human fibroblasts, nucleolar size correlates positively with donor age [17]. Furthermore, in *C. elegans*, knockdown of fibrillarin reduces nucleolar size and extends lifespan, supporting a causal link between nucleolar expansion and aging [16,18].

The target of rapamycin (TOR) signaling pathway plays a central role in regulating nucleolar size during aging [19,20]. Age-related increases in nutrient and growth factor signaling, combined with mitochondrial dysfunction, can contribute to aberrant TOR activation, which in turn promotes rDNA transcription and ribosome biogenesis. Pharmacological inhibition of TOR by rapamycin reduces nucleolar size and extends lifespan in multiple model organisms [16,19,20]. These findings raise the possibility that TBI may increase nucleolar size by accelerating age-related processes. In support of this idea, treatment with rapamycin improves various pathological outcomes of TBI in rodents [21–28].

To investigate whether TBI affects nucleolar size in the brain, we used a *Drosophila melanogaster* model in which closed-head injury and associated polytrauma are induced with a High-Impact Trauma (HIT) device [29,30]. We previously showed that TBI outcomes in flies are strongly age-dependent and engage conserved aging pathways [29,31–33]. Specifically, early mortality (death within 24 h of injury) increased with age at the time of injury and correlated with the median lifespan of different inbred fly lines [31,32]. Flies from longer-lived lines had lower early mortality than shorter-lived lines when injured at the same chronological age. Similarly, genetic and environmental interventions that extended lifespan also reduced early mortality after TBI. Additionally, we found that TBI in flies triggered innate immune system activation [32,34,35] and increased intestinal permeability [31,36], which are markers of impending death during normal aging [37], and caused severe neurodegeneration when older flies were injured [29]. These findings establish Drosophila as a powerful model for studying age-dependent TBI outcomes.

Using immunofluorescence microcopy, we found that TBI in young flies (3−6 days old) caused a substantial increase in brain nucleolar size at 1 day post-injury that was maintained over the following 40 days. In contrast, injury of older flies (26−29 or 47−50 days old) resulted in little or no nucleolar enlargement at 1 day after injury and smaller nucleoli than uninjured controls by 5 days post-injury. Furthermore, treatment of young flies with rapamycin or RapaLink-1, another TOR inhibitor, reduced early mortality after TBI. These findings support the idea that TBI accelerates aging-related increases in ribosome biogenesis, contributing to adverse outcomes.

## Results and discussion

### TBI rapidly increases nucleolar size

**Fig 1:**
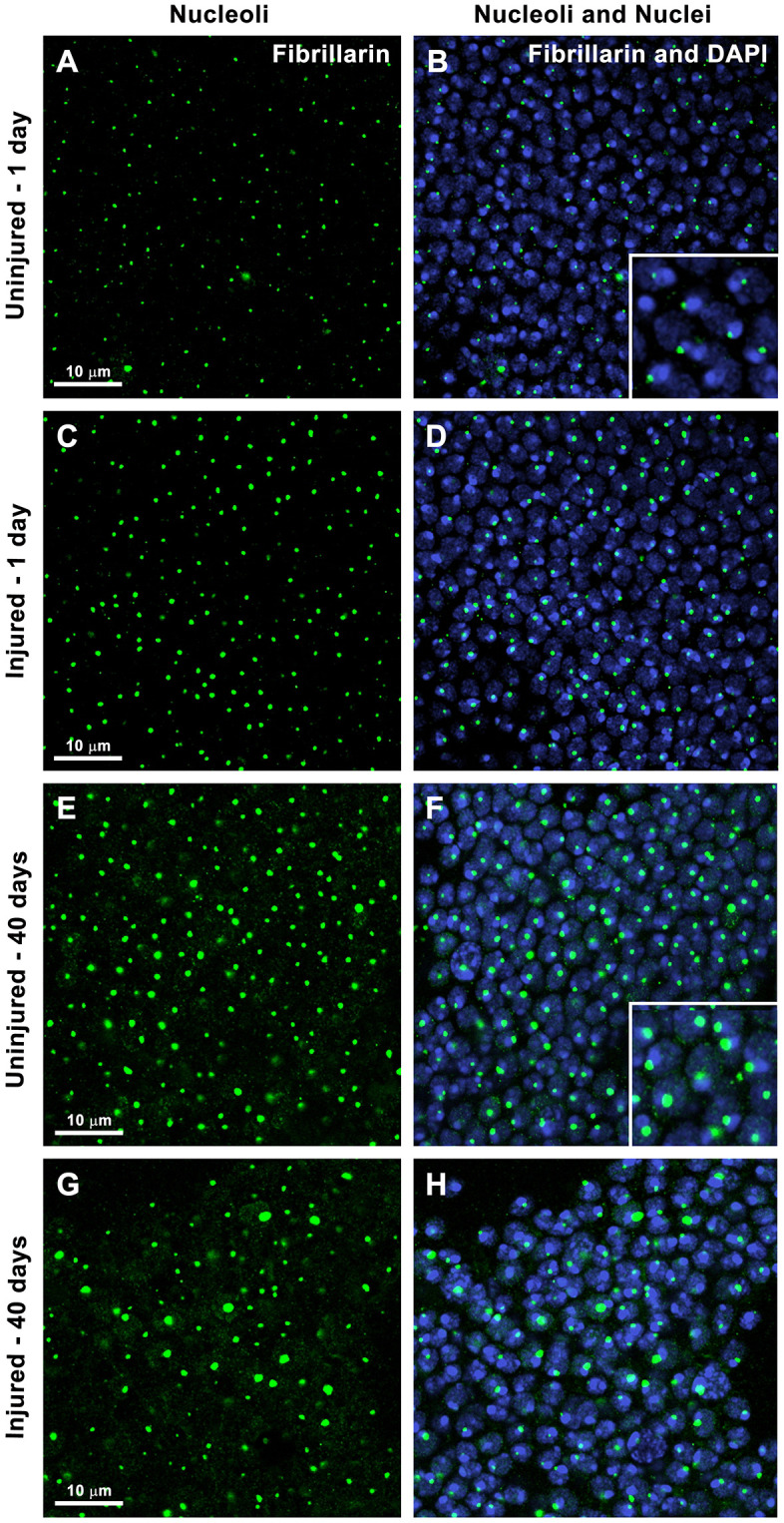
TBI and aging both increase nucleolar size in Drosophila brain. Representative confocal microscopy images of the central brain from uninjured (A, B, E, and F) and injured (C, D, G, and H) mixed-sex, 3-6 day old, *w*^*1118*^ flies at 1 day (A-D) and 40 days (E-H) post-injury. (B and F) Insets show magnified views, highlighting nucleoli located next to bright DAPI-stained spots. Nuclei are labeled with DAPI (blue) and nucleoli are labeled with fibrillarin antibody (green).

To investigate nucleolar dynamics following TBI, we used confocal immunofluorescence microscopy to assess nucleolar size in the central brain of injured and uninjured adult flies. These experiments used a standard laboratory fly strain, *w*^*1118*^. Mixed-sex flies (1:1, female:male) were injured at 3−6 days old using a HIT device; and age-matched, uninjured flies served as controls. Consistent with previous studies, injury by the HIT device caused 25% early mortality in this strain [29,31,32]. Brains from surviving flies and uninjured controls were stained with an antibody to fibrillarin to visualize nucleoli and DAPI (4’,6-diamidino-2-phenylindole) to label nuclear DNA in neurons and glia (Fig 1).

In both injured and uninjured brains at 1 day post-injury, each nucleus contained a single nucleolus positioned adjacent to or partially embedded within a brightly DAPI-stained region (Figs 1B (inset) and 1D). Nucleoli in Drosophila form around multicopy rDNA arrays located in the heterochromatic regions of the *X* and *Y* chromosomes [38]. Because heterochromatin is rich in A-T base pairs, it binds DAPI with high affinity and appears strongly fluorescent [39]. In Drosophila, rDNA arrays on the sex chromosomes are typically paired, resulting in the formation of a single nucleolus per nucleus [38,40]. Thus, the data in Fig 1 are consistent with previous reports of nucleolar organization in Drosophila somatic cells.

**Fig 2:**
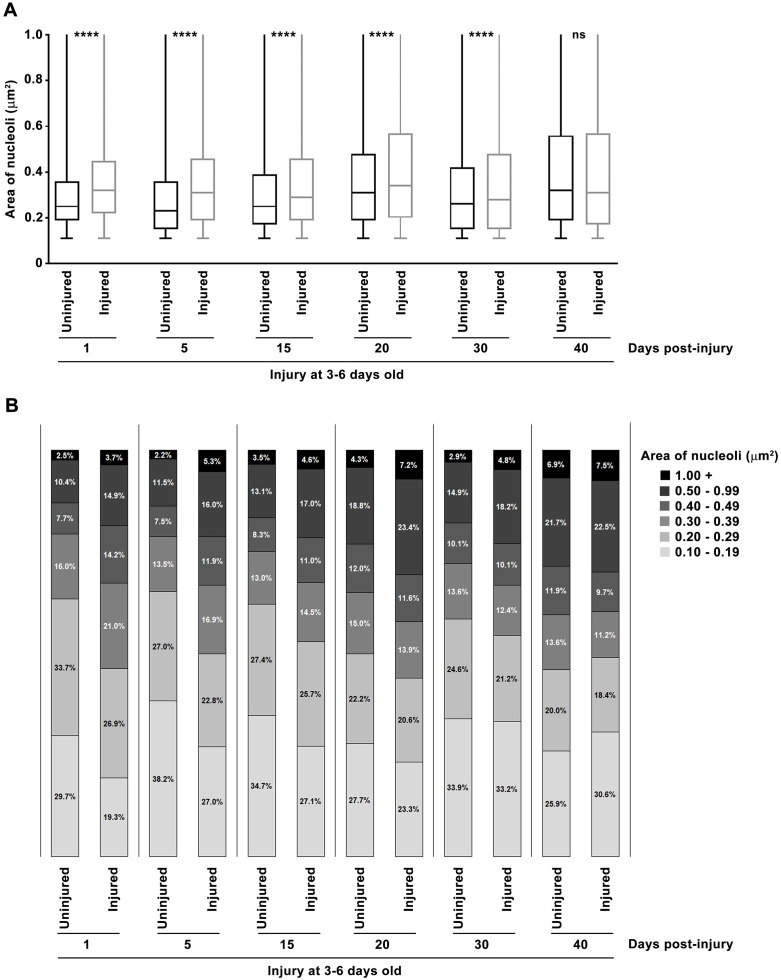
TBI and aging promote nucleolar expansion. Nucleolar area was measured by fibrillarin staining of dissected brains from 3-6 day old, mixed-sex, *w*^*1118*^ flies that were either uninjured or injured. For each condition and time point, at least 11 brains and 7,000 nucleoli were examined. (A) Nucleolar area is shown as box-and-whisker plots, with the median (horizonal line), interquartile range (box), and minimum and maximum values (whiskers). p-values reflect comparisons between injured and uninjured flies using one-way analysis of variance (ANOVA) with Tukey’s multiple comparison test. ns = not significant, ****p < 0.0001. (B) Distribution of nucleolar areas, shown as the percent of nucleoli falling within each of five size ranges (see figure key).

Visual inspection of images suggested that nucleoli were larger in injured flies compared with uninjured controls at 1 day after injury (compare Fig 1A and 1C). Quantitative analysis of at least 11 brains and 7,000 nuclei for each condition confirmed this observation: the median nucleolar area was significantly larger in injured flies (Fig 2A). Injury increased the median nucleolar area by 28.0%, from 0.25 μm^2^ in uninjured flies to 0.32 μm^2^ in injured flies. This shift was driven by a reduced proportion of small nucleoli (≤0.29 μm^2^) and an increased proportion of larger nucleoli (≥0.3 μm^2^) (Fig 2B). These findings suggest that TBI stimulates ribosome biogenesis, leading to nucleolar enlargement. However, it remains to be determined whether rRNA and ribosomal protein synthesis, and thus overall translational capacity, are elevated. Further work is also needed to identity which cell types exhibit enlarged nucleoli. Given that 17.2% of cells showed increased nucleolar size at 1 day post-injury (Fig 1B), whereas only 10% of brain cells are glia [41], the effect likely occurs in both neurons and glia or exclusively in neurons. To our knowledge, this is the first report showing that TBI alters nucleolar size, adding to the growing evidence TBI elicits cellular responses similar to those observed during normal aging [6,7,9–11].

### TBI accelerates age-associated nucleolar enlargement

To investigate the long-term effect of TBI on nucleolar dynamics, we used immunofluorescence microscopy of fibrillarin to examine nucleoli at 5, 15, 20, 30, and 40 days post-injury. In uninjured control flies, nucleoli were visibly larger in older flies (43–46 day old, *i.e*., 3–6 day old flies aged 40 days, Fig 1E) compared to young flies (4–7 day old, *i.e*., 3–6 day old flies aged 1 day, Fig 1A). This visual observation was supported by quantitative analysis: in uninjured flies, the median nucleolar area increased by 28.0%, from 0.25 μm^2^ at 1 day to 0.32 μm^2^ at 40 days (Fig 2A). This increase was non-linear, with decreases between days 1–5 and 20–30. Thus, the nucleolar enlargement seen 1 day after injury is similar in magnitude to the age-related increase that occurs over 40 days in uninjured flies. These findings raise the possibility that injured cells undergo senescence, a state marked by nucleolar enlargement [42] and induced by age-associated stresses as well as by increased rRNA transcription or decreased rRNA processing [43,44].

As flies aged, the difference in nucleolar area between injured and uninjured flies diminished. At 1 day post-injury, the proportion of nucleoli with an area ≥0.3 μm^2^ was 17.2% higher in injured flies than in uninjured controls. This difference steadily declined to 15.4, 9.2, 6.0, 4.0, and −3.1% at 5, 15, 20, 30, and 40 days post-injury, respectively (Fig 2B). Since the median nucleolar area remained relatively stable in injured flies over time (Fig 2A), the narrowing gap between injured and uninjured flies is attributable to the age-dependent increase in nucleolar size in uninjured flies.

### TBI and aging increase cell-to-cell variation in nucleolar size

In both injured and uninjured flies, the area of nucleoli ranged from 0.1 to >1.0 μm^2^ (Fig 2A), reflecting a combination of biological heterogeneity and imaging effects – nucleoli appear largest when sectioned through the center and smaller near the surface. One day post-injury, the interquartile range (IQR) of nucleolar size (25^th^ to the 75^th^ percentile) was narrower in uninjured flies (0.19–0.36 μm^2^) than in injured flies (0.22–0.45 μm^2^), suggesting that injury increases cell-to-cell variation in nucleolar size (see boxes in Fig 2A). Aging showed a similar effect: visual inspection (Fig 1A vs. 1E; 1C vs. 1G) and IQR analysis (Fig 2A) revealed that variation in nucleolar size widened with age. In uninjured flies, the IQR expanded from 0.19–0.36 μm^2^ at 1 day to 0.19–0.56 μm^2^ at 40 days; in injured flies, it widened from 0.22–0.45 μm^2^ at 1 day to 0.17–0.57 μm^2^ at 40 days. These findings suggest that both TBI and aging activate signaling pathways that drive nucleolar enlargement, but the extent of enlargement differs among brain cells, possibly due to cell-to-cell variation in gene expression [45,46].

### Age at the time of TBI influences the nucleolar response to injury

**Fig 3:**
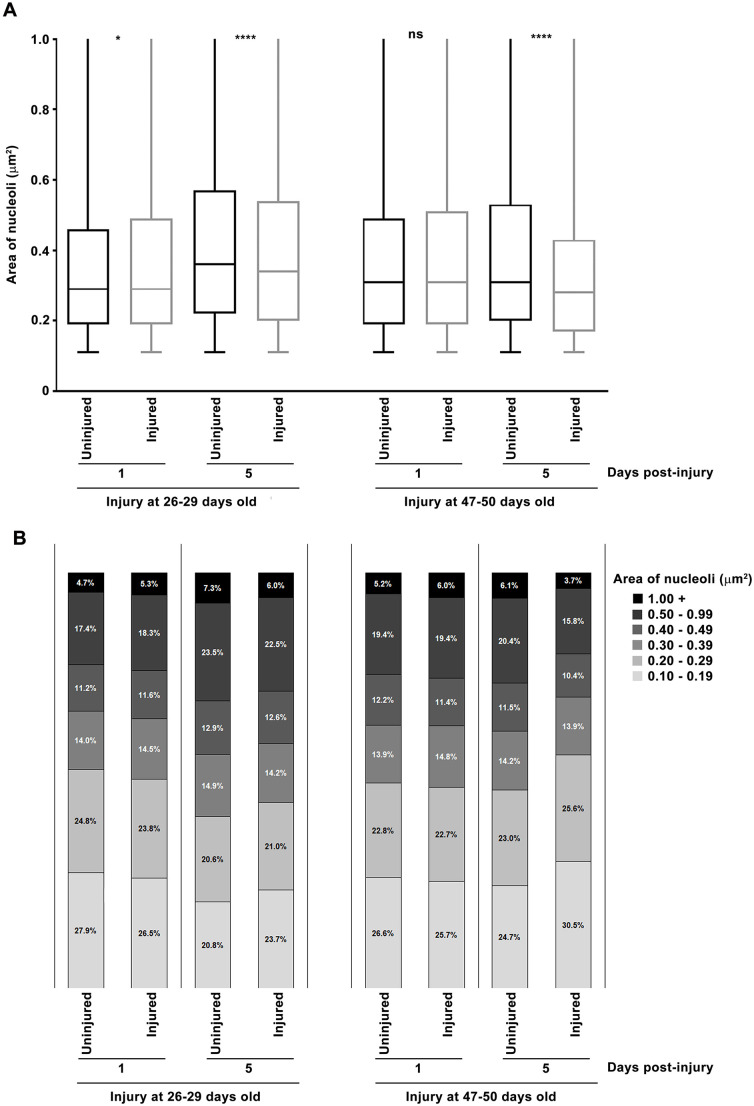
TBI has minimal acute effects on nucleolar expansion in older flies. Nucleolar area was determined by fibrillarin staining of dissected brains from mixed-sex, *w*^*1118*^ flies that were either uninjured or injured at 26-29 or 47-50 days old and aged 1 or 5 days. For each condition and time point, at least 11 brains and 7,000 nuclei were analyzed. (A) Nucleolar area is shown as box-and-whiskers plots, displaying the median (horizonal line), interquartile range (box), and minimum and maximum values (whiskers). p-values reflect comparisons between injured and uninjured flies using one-way ANOVA with Tukey’s multiple comparison test. ns = not significant, *p < 0.05, ****p < 0.0001. (B) Distribution of nucleolar areas, shown as the percent of nucleoli falling within each of five size ranges (see figure key).

To investigate how aging influences the effect of TBI on nucleolar dynamics, we repeated the 1 and 5 day post-injury analyses using flies injured at 26–29 or 47–50 days old. Consistent with our prior studies [29,31–33], early mortality increased with age at the time of injury: 30% for 26–29 day old flies and 45% for 47–50 day old flies. At 1 day post-injury, both 26–29 and 47–50 day old flies showed no difference in median nucleolar area between injured flies and uninjured controls (Fig 3A). This contrasts with the 28.0% increase observed in flies injured at 3–6 day old (Fig 2A). At 5 days post-injury, median nucleolar area was significantly reduced in both older groups – by 5.6% in 26–29 day old flies and by 9.7% in 47–50 day old flies (Fig 3A) – whereas nucleoli in injured 3–6 day old flies were 34.8% larger (Fig 2A). These reductions in older flies were associated with an increased proportion of nucleoli ≤0.29 μm^2^ and a corresponding decrease in those ≥0.3 μm^2^ (Fig 3B). These findings suggest that aging activates the molecular pathways that regulate nucleolar size to their maximum capacity, leaving no additional effect for TBI to exert.

TBI-induced changes in nucleolar dynamics may contribute to downstream age-related pathologies, including mitochondrial dysfunction, DNA damage, cellular senescence, and neurodegeneration. Ribosome biogenesis, a highly energy- and resource-demanding process, can strain mitochondrial capacity, impairing function [47]. In addition, expansion of the nucleolus beyond a critical size can compromise nucleolar integrity, leading to DNA damage through influx of the homologous recombination repair protein Rad52 and aberrant recombination [22]. Excessive rDNA transcription without coordinated ribosome biogenesis can drive cells into senescence [44]. Finally, reduced nucleolar size, as occurred days after injury of older flies (Fig 3), is seen in neurodegenerative diseases like Alzheimer’s and Parkinson’s [13,48–51].

### Inhibition of the TOR pathway reduces early mortality following TBI

**Fig 4:**
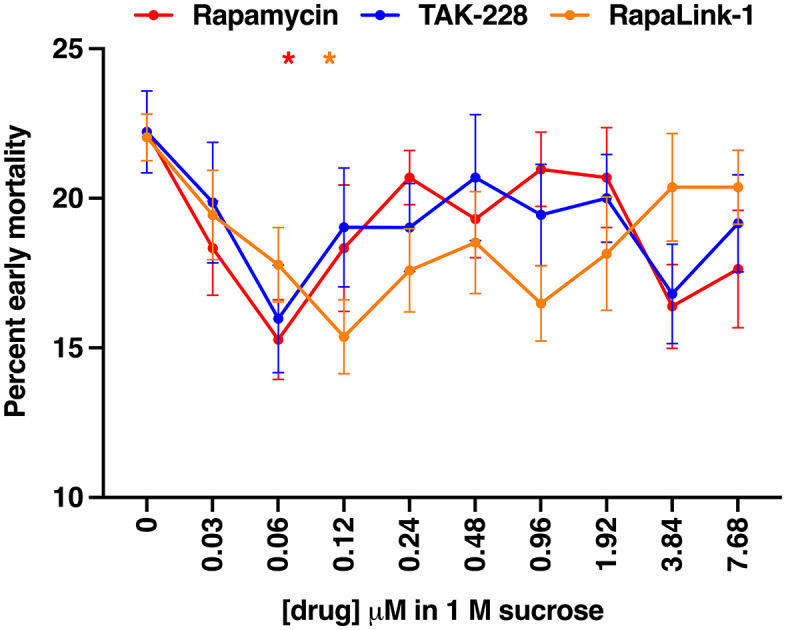
TOR inhibitors reduce early mortality after TBI in a dose-dependent manner. Mixed-sex, *w*^*1118*^ flies (0-7 days old) were fed rapamycin, TAK-228, or RapaLink-1 in 1 M sucrose following injury. Early mortality was measured as the percent of flies that died within 24 h post-injury. Data represent the mean ± standard error of the mean (SEM) from 12 replicates (60 flies each) for rapamycin, 12 replicates for TAK-228, and 9 replicates for RapaLink-1. Statistical comparisons of each drug treatment were performed using one-way ANOVA with Dunnett’s multiple comparison test; *p < 0.05.

To test whether nucleolar expansion contributes to TBI outcomes, we assessed the effect of TOR pathway inhibition on early mortality. Mixed-sex, *w*^*1118*^ flies (0–7 days old) were injured and then fed the TOR inhibitors rapamycin, TAK-228, or RapaLink-1 delivered in 1 M sucrose across a 256-fold concentration range (0.03–7.68 μM). We have previously shown that feeding flies 1 M sucrose results in the same percent early mortality following TBI as flies fed standard fly food [31]. TAK-228, also known as MLN0128 and INK128, is an ATP-competitive TOR kinase inhibitor [52,53], and RapaLink-1 is a bifunctional molecule that chemically links rapamycin to TAK-228 [54]. Rapamycin and RapaLink-1 significantly reduced early mortality at 0.06 μM and 0.12 μM, respectively, with reductions of 30.6% and 30.2% (Fig 4). TAK-228 also showed a near-significant effect at 0.06 μM (*p* = 0.079). These findings suggest that TOR-dependent nucleolar expansion contributes to TBI pathogenesis. Because rapamycin and RapaLink-1 primarily inhibit mechanistic target of rapamycin complex 1 (mTORC1) rather than mTORC2 in flies, our findings further suggest that early mortality following TBI is mediated by mTORC1 [55]. However, although reduced early mortality following TOR inhibition is consistent with TOR-dependent nucleolar expansion, definitive proof will require directly measuring nucleolar size in flies treated with TOR inhibitors. Such measurements are necessary to establish a causal link between nucleolar enlargement and early mortality.

Mechanisms linking nucleolar expansion to TBI pathogenesis remain unclear. One possibility is that increased ribosome biogenesis elevates protein synthesis beyond the capacity of protein folding and quality control systems, resulting in protein aggregation and endoplasmic reticulum stress [56] – both observed after TBI [57,58]. Another is that the high energy demand of ribosome production [59] depletes energy in neurons already impaired by mitochondrial dysfunction [60,61], reducing their ability to maintain ion gradients and detoxify reactive oxygen species. Alternatively, nucleolar expansion may arise from an imbalance between ribosomal proteins and rRNA, leading to accumulation of unprocessed rRNA, ribosomal precursors, or orphan ribosomal proteins [62,63]. This can trigger nucleolar stress and cellular toxicity, effects that can be mitigated by TOR inhibition [64].

In summary, we found that TBI induced rapid nucleolar enlargement that persisted for weeks in young flies but was absent in older ones, indicating an age-dependent response (Figs 1–3). Both TBI and aging also increased cell-to-cell variation in nucleolar size (Figs 1 and 2). Notably, inhibition of TOR signaling – a key regulator of nucleolar size – reduced early mortality after TBI (Fig 4). These findings suggest that nucleolar enlargement contributes to TBI-related adverse outcomes and highlight Drosophila as a powerful model for dissecting the mechanisms that link nucleolar dynamics to TBI pathophysiology.

## Materials and methods

### Fly culturing and TBI

Flies were maintained at 25°C on a standard fly food containing cornmeal, molasses, and yeast [65]. The *w*^*1118*^ strain has been maintained in our lab for many years. Flies were collected at 0–3 days post-eclosion, aged as indicated, and subjected to TBI in groups of 60 using a HIT device (4 strikes, spaced 5 min apart), following the protocol described in Katzenberger *et al*., 2013 [29]. Control (uninjured) flies were handled identically but were not subjected to strikes. After treatment, both injured and uninjured flies were transferred to fresh food vials and maintained at 25°C until analysis. Early mortality was calculated as the percent of flies that died within 24 h post-injury, adjusted by subtracting the percent mortality in the uninjured group. Flies were considered dead if they remained immobile following gentle agitation.

### Confocal immunofluorescence microscopy and quantitation of nucleolar area

Brains were dissected under a light microscope in a drop of fresh, ice cold 4% formaldehyde on a Sylgard plate (Dow Inc.) with #5 Dumont forceps (Fine Science Tools) and transferred to an Eppendorf tube containing 1 ml 4% formaldehyde on ice for approximately 30 min. Brains were washed 3 x 20 min in 1.5 ml 1X phosphate buffered saline (PBS) with 0.3% Triton-X (PBS-T), blocked in 500 μl PBS-T with 5% normal goat serum (Sigma) overnight at 4°C. Block was removed and 1.5 ml fibrillarin antibody (1:500 in PBS-T, 1:100, Cytoskeleton (AFB01)) was added and incubated overnight at 4°C. Brains were washed 3 x 20 min in 1.5 ml PBS-T at room temperature. Fluorescently labeled secondary antibody (α-mouse Alexa Fluor 488, 1:500 in PBS-T, Invitrogen) was added and incubated overnight at 4°C. Brains were washed 3 x in 1.5 ml PBS-T at room temperature. The first wash (10 min) contained DAPI (1 mg/mL in H_2_O, diluted 1:1000 in PBS-T, Santa Cruz Biotechnology), and the second and third washes (10 min each) were only PBS-T. During washes and incubations, tubes were placed in a light tight box and agitated. Brains were mounted in Vectashield (Vector Laboratories) and imaged at 100x magnification on a Nikon A1R-SI+ confocal microscope (Optical Imaging Core, University of Wisconsin, Madison, WI).

Nikon Elements Imaging Software (Optical Imaging Core, University of Wisconsin, Madison, WI) was used to determine the area of individual nucleoli in the central brain. 100x single channel confocal images were used for this analysis of at least 11 brains and 7,000 nucleoli. In the 488 channel, fibrillarin-stained nucleoli were selected through thresholding to create a binary mask. The software calculated the area based on the number of pixels within each focus in the binary mask, converting pixels to calibrated units (μm^2^). Approximately half of the brains were imaged from the posterior side and the other half from the anterior side. Nucleoli were sorted by area using Microsoft Excel, and those >0.1 μm^2^ were counted. GraphPad Prism (version 10.5.0) was used for graphing and statistical analyses of the data in Figs 2 and 3.

### Drug treatment

Injured and uninjured, mixed-sex, 0–7 day old, *w*^*1118*^ flies were transferred to vials containing a filter paper disc at the bottom absorbed with 200 μl of 1 M sucrose or rapamycin (Sigma), TAK-228, or RapaLink-1 (TAK-228 and RapaLink-1 were supplied by Douglas Wassarman and Kevan Shokat, University of California-San Francisco) at the indicated concentrations in 1 M sucrose (Fig 4). For each concentration and condition, 12, 12, and 9 independent samples of 60 flies were examined for rapamycin, TAK-228, and RapaLink-1, respectively. GraphPad Prism (version 10.5.0) was used for graphing and statistical analyses of the data in Fig 4.

## Supporting information

S1 Data Effects of TBI on nucleolar size in flies of different ages. The first and second tabs show the primary data used in Figs 2 and 3, respectively. Columns indicate experimental conditions and rows are sizes of individual nucleoli in μm^2^.
(XLSX)

S2 Data Effects of TOR inhibitors on early mortality following TBI. The first, second, and third tabs show the primary early mortality data for rapamycin, TAK-228, and Rapalink-1, respectively, used in Fig 4. Data in rows 2–11 represent the number of dead flies out of 60 for each of the 9 or 12 replicate experiments under each drug concentration. Data in rows 14–23 represent the percent early mortality.
(XLSX)

## References

[1] Taylor CA, Bell JM, Breiding MJ, Xu L. Traumatic brain injury-related emergency department visits, hospitalizations, and deaths - United States, 2007 and 2013. MMWR Surveill Summ. 2017;66(9):1–16. doi: 10.15585/mmwr.ss6609a1 28301451 PMC5829835

[2] Hyder AA, Wunderlich CA, Puvanachandra P, Gururaj G, Kobusingye OC. The impact of traumatic brain injuries: a global perspective. NeuroRehabilitation. 2007;22(5):341–53. doi: 10.3233/nre-2007-22502 18162698

[3] Masel BE, DeWitt DS. Traumatic brain injury: a disease process, not an event. J Neurotrauma. 2010;27(8):1529–40. doi: 10.1089/neu.2010.1358 20504161

[4] Blennow K, Hardy J, Zetterberg H. The neuropathology and neurobiology of traumatic brain injury. Neuron. 2012;76(5):886–99. doi: 10.1016/j.neuron.2012.11.021 23217738

[5] Prins M, Greco T, Alexander D, Giza CC. The pathophysiology of traumatic brain injury at a glance. Dis Model Mech. 2013;6(6):1307–15. doi: 10.1242/dmm.011585 24046353 PMC3820255

[6] Lu Y, Jarrahi A, Moore N, Bartoli M, Brann DW, Baban B, et al. Inflammaging, cellular senescence, and cognitive aging after traumatic brain injury. Neurobiol Dis. 2023;180:106090. doi: 10.1016/j.nbd.2023.106090 36934795 PMC10763650

[7] Smith DH, Johnson VE, Stewart W. Chronic neuropathologies of single and repetitive TBI: substrates of dementia? Nat Rev Neurol. 2013;9(4):211–21. doi: 10.1038/nrneurol.2013.29 23458973 PMC4513655

[8] López-Otín C, Blasco MA, Partridge L, Serrano M, Kroemer G. Hallmarks of aging: an expanding universe. Cell. 2023;186(2):243–78. doi: 10.1016/j.cell.2022.11.001 36599349

[9] Barker S, Paul BD, Pieper AA. Increased risk of aging-related neurodegenerative disease after traumatic brain injury. Biomedicines. 2023;11(4):1154. doi: 10.3390/biomedicines11041154 37189772 PMC10135798

[10] Kumar Sahel D, Kaira M, Raj K, Sharma S, Singh S. Mitochondrial dysfunctioning and neuroinflammation: recent highlights on the possible mechanisms involved in Traumatic Brain Injury. Neurosci Lett. 2019;710:134347. doi: 10.1016/j.neulet.2019.134347 31229625

[11] Goyal L, Singh S. Neurological manifestations following traumatic brain injury: role of behavioral, neuroinflammation, excitotoxicity, Nrf-2 and nitric oxide. CNS Neurol Disord Drug Targets. 2025;24(1):47–59. doi: 10.2174/0118715273318552240708055413 39082170

[12] Matos-Perdomo E, Machín F. Nucleolar and ribosomal DNA Structure under Stress: yeast lessons for aging and cancer. Cells. 2019;8(8):779. doi: 10.3390/cells8080779 31357498 PMC6721496

[13] Hetman M, Pietrzak M. Emerging roles of the neuronal nucleolus. Trends Neurosci. 2012;35(5):305–14. doi: 10.1016/j.tins.2012.01.002 22305768 PMC3348388

[14] Cerqueira AV, Lemos B. Ribosomal DNA and the nucleolus as keystones of nuclear architecture, organization, and function. Trends Genet. 2019;35(10):710–23. doi: 10.1016/j.tig.2019.07.011 31447250 PMC8487316

[15] Neumüller RA, Gross T, Samsonova AA, Vinayagam A, Buckner M, Founk K, et al. Conserved regulators of nucleolar size revealed by global phenotypic analyses. Sci Signal. 2013;6(289):ra70. doi: 10.1126/scisignal.2004145 23962978 PMC3964804

[16] Tiku V, Jain C, Raz Y, Nakamura S, Heestand B, Liu W, et al. Small nucleoli are a cellular hallmark of longevity. Nat Commun. 2017;8:16083. doi: 10.1038/ncomms16083 28853436 PMC5582349

[17] Buchwalter A, Hetzer MW. Nucleolar expansion and elevated protein translation in premature aging. Nat Commun. 2017;8(1):328. doi: 10.1038/s41467-017-00322-z 28855503 PMC5577202

[18] Tiku V, Antebi A. Nucleolar function in lifespan regulation. Trends Cell Biol. 2018;28(8):662–72. doi: 10.1016/j.tcb.2018.03.007 29779866

[19] Mannick JB, Lamming DW. Targeting the biology of aging with mTOR inhibitors. Nat Aging. 2023;3(6):642–60. doi: 10.1038/s43587-023-00416-y 37142830 PMC10330278

[20] Kennedy BK, Lamming DW. The mechanistic target of rapamycin: the grand conductor of metabolism and aging. Cell Metab. 2016;23(6):990–1003. doi: 10.1016/j.cmet.2016.05.009 27304501 PMC4910876

[21] Erlich S, Alexandrovich A, Shohami E, Pinkas-Kramarski R. Rapamycin is a neuroprotective treatment for traumatic brain injury. Neurobiol Dis. 2007;26(1):86–93. doi: 10.1016/j.nbd.2006.12.003 17270455

[22] Campolo M, Casili G, Lanza M, Filippone A, Cordaro M, Ardizzone A, et al. The inhibition of mammalian target of rapamycin (mTOR) in improving inflammatory response after traumatic brain injury. J Cell Mol Med. 2021;25(16):7855–66. doi: 10.1111/jcmm.16702 34245104 PMC8358860

[23] Chen Y, Meng J, Xu Q, Long T, Bi F, Chang C, et al. Rapamycin improves the neuroprotection effect of inhibition of NLRP3 inflammasome activation after TBI. Brain Res. 2019;1710:163–72. doi: 10.1016/j.brainres.2019.01.005 30615886

[24] Song Q, Xie D, Pan S, Xu W. Rapamycin protects neurons from brain contusion‑induced inflammatory reaction via modulation of microglial activation. Mol Med Rep. 2015;12(5):7203–10. doi: 10.3892/mmr.2015.4407 26458361 PMC4626160

[25] Nikolaeva I, Crowell B, Valenziano J, Meaney D, D’Arcangelo G. Beneficial effects of early mTORC1 inhibition after traumatic brain injury. J Neurotrauma. 2016;33(2):183–93. doi: 10.1089/neu.2015.3899 26122481 PMC4722609

[26] Ding K, Wang H, Wu Y, Zhang L, Xu J, Li T, et al. Rapamycin protects against apoptotic neuronal death and improves neurologic function after traumatic brain injury in mice via modulation of the mTOR-p53-Bax axis. J Surg Res. 2015;194(1):239–47. doi: 10.1016/j.jss.2014.09.026 25438952

[27] Guo D, Zeng L, Brody DL, Wong M. Rapamycin attenuates the development of posttraumatic epilepsy in a mouse model of traumatic brain injury. PLoS One. 2013;8(5):e64078. doi: 10.1371/journal.pone.0064078 23691153 PMC3653881

[28] Park J, Zhang J, Qiu J, Zhu X, Degterev A, Lo EH, et al. Combination therapy targeting Akt and mammalian target of rapamycin improves functional outcome after controlled cortical impact in mice. J Cereb Blood Flow Metab. 2012;32(2):330–40. doi: 10.1038/jcbfm.2011.131 21934697 PMC3272599

[29] Katzenberger RJ, Loewen CA, Wassarman DR, Petersen AJ, Ganetzky B, Wassarman DA. A Drosophila model of closed head traumatic brain injury. Proc Natl Acad Sci U S A. 2013;110(44):E4152-9. doi: 10.1073/pnas.1316895110 24127584 PMC3816429

[30] Katzenberger RJ, Loewen CA, Bockstruck RT, Woods MA, Ganetzky B, Wassarman DA. A method to inflict closed head traumatic brain injury in Drosophila. J Vis Exp. 2015;(100):e52905. doi: 10.3791/52905 26168076 PMC4544997

[31] Katzenberger RJ, Chtarbanova S, Rimkus SA, Fischer JA, Kaur G, Seppala JM, et al. Death following traumatic brain injury in Drosophila is associated with intestinal barrier dysfunction. Elife. 2015;4:e04790. doi: 10.7554/eLife.04790 25742603 PMC4377547

[32] Katzenberger RJ, Ganetzky B, Wassarman DA. Age and diet affect genetically separable secondary injuries that cause acute mortality following traumatic brain injury in Drosophila. G3 (Bethesda). 2016;6(12):4151–66. doi: 10.1534/g3.116.036194 27754853 PMC5144983

[33] Katzenberger RJ, Ganetzky B, Wassarman DA. Lissencephaly-1 mutations enhance traumatic brain injury outcomes in Drosophila. Genetics. 2023;223(3):iyad008. doi: 10.1093/genetics/iyad008 36683334 PMC9991514

[34] Swanson LC, Trujillo EA, Thiede GH, Katzenberger RJ, Shishkova E, Coon JJ, et al. Survival following traumatic brain injury in *Drosophila* is increased by heterozygosity for a mutation of the NF-κB innate immune response transcription factor relish. Genetics. 2020;216(4):1117–36. doi: 10.1534/genetics.120.303776 33109529 PMC7768241

[35] Swanson LC, Rimkus SA, Ganetzky B, Wassarman DA. Loss of the antimicrobial peptide metchnikowin protects against traumatic brain injury outcomes in *Drosophila melanogaster*. G3 (Bethesda). 2020;10(9):3109–19. doi: 10.1534/g3.120.401377 32631949 PMC7466987

[36] Scharenbrock AR, Katzenberger RJ, Fischer MC, Ganetzky B, Wassarman DA. Beta-blockers reduce intestinal permeability and early mortality following traumatic brain injury in *Drosophila*. MicroPubl Biol. 2021;2021:10.17912/micropub.biology.000461. doi: 10.17912/micropub.biology.000461 34723144 PMC8553408

[37] Rera M, Clark RI, Walker DW. Intestinal barrier dysfunction links metabolic and inflammatory markers of aging to death in Drosophila. Proc Natl Acad Sci U S A. 2012;109(52):21528–33. doi: 10.1073/pnas.1215849110 23236133 PMC3535647

[38] Greil F, Ahmad K. Nucleolar dominance of the Y chromosome in Drosophila melanogaster. Genetics. 2012;191(4):1119–28. doi: 10.1534/genetics.112.141242 22649076 PMC3415996

[39] Kapuscinski J. DAPI: a DNA-specific fluorescent probe. Biotech Histochem. 1995;70(5):220–33. doi: 10.3109/10520299509108199 8580206

[40] Boamah EK, Kotova E, Garabedian M, Jarnik M, Tulin AV. Poly(ADP-Ribose) polymerase 1 (PARP-1) regulates ribosomal biogenesis in Drosophila nucleoli. PLoS Genet. 2012;8(1):e1002442. doi: 10.1371/journal.pgen.1002442 22242017 PMC3252306

[41] Kremer MC, Jung C, Batelli S, Rubin GM, Gaul U. The glia of the adult Drosophila nervous system. Glia. 2017;65(4):606–38. doi: 10.1002/glia.23115 28133822 PMC5324652

[42] Lewinska A, Miedziak B, Kulak K, Molon M, Wnuk M. Links between nucleolar activity, rDNA stability, aneuploidy and chronological aging in the yeast Saccharomyces cerevisiae. Biogerontology. 2014;15(3):289–316. doi: 10.1007/s10522-014-9499-y 24711086 PMC4019837

[43] Nishimura K, Kumazawa T, Kuroda T, Katagiri N, Tsuchiya M, Goto N, et al. Perturbation of ribosome biogenesis drives cells into senescence through 5S RNP-mediated p53 activation. Cell Rep. 2015;10(8):1310–23. doi: 10.1016/j.celrep.2015.01.055 25732822

[44] Morlot S, Song J, Léger-Silvestre I, Matifas A, Gadal O, Charvin G. Excessive rDNA transcription drives the disruption in nuclear homeostasis during entry into senescence in budding yeast. Cell Rep. 2019;28(2):408-422.e4. doi: 10.1016/j.celrep.2019.06.032 31291577

[45] Chien J-F, Liu H, Wang B-A, Luo C, Bartlett A, Castanon R, et al. Cell-type-specific effects of age and sex on human cortical neurons. Neuron. 2024;112(15):2524–39.e5. doi: 10.1016/j.neuron.2024.05.013 38838671

[46] Mendenhall AR, Martin GM, Kaeberlein M, Anderson RM. Cell-to-cell variation in gene expression and the aging process. Geroscience. 2021;43(1):181–96. doi: 10.1007/s11357-021-00339-9 33595768 PMC8050212

[47] Corman A, Sirozh O, Lafarga V, Fernandez-Capetillo O. Targeting the nucleolus as a therapeutic strategy in human disease. Trends Biochem Sci. 2023;48(3):274–87. doi: 10.1016/j.tibs.2022.09.006 36229381

[48] Hetman M, Slomnicki LP. Ribosomal biogenesis as an emerging target of neurodevelopmental pathologies. J Neurochem. 2019;148(3):325–47. doi: 10.1111/jnc.14576 30144322 PMC6347560

[49] Iacono D, O’Brien R, Resnick SM, Zonderman AB, Pletnikova O, Rudow G, et al. Neuronal hypertrophy in asymptomatic Alzheimer disease. J Neuropathol Exp Neurol. 2008;67(6):578–89. doi: 10.1097/NEN.0b013e3181772794 18520776 PMC2518071

[50] Rieker C, Engblom D, Kreiner G, Domanskyi A, Schober A, Stotz S, et al. Nucleolar disruption in dopaminergic neurons leads to oxidative damage and parkinsonism through repression of mammalian target of rapamycin signaling. J Neurosci. 2011;31(2):453–60. doi: 10.1523/JNEUROSCI.0590-10.2011 21228155 PMC6623444

[51] Kreiner G, Bierhoff H, Armentano M, Rodriguez-Parkitna J, Sowodniok K, Naranjo JR, et al. A neuroprotective phase precedes striatal degeneration upon nucleolar stress. Cell Death Differ. 2013;20(11):1455–64. doi: 10.1038/cdd.2013.66 23764776 PMC3792439

[52] Hsieh AC, Liu Y, Edlind MP, Ingolia NT, Janes MR, Sher A, et al. The translational landscape of mTOR signalling steers cancer initiation and metastasis. Nature. 2012;485(7396):55–61. doi: 10.1038/nature10912 22367541 PMC3663483

[53] Rodrik-Outmezguine VS, Okaniwa M, Yao Z, Novotny CJ, McWhirter C, Banaji A, et al. Overcoming mTOR resistance mutations with a new-generation mTOR inhibitor. Nature. 2016;534(7606):272–6. doi: 10.1038/nature17963 27279227 PMC4902179

[54] Fan Q, Aksoy O, Wong RA, Ilkhanizadeh S, Novotny CJ, Gustafson WC, et al. A kinase inhibitor targeted to mTORC1 drives regression in glioblastoma. Cancer Cell. 2017;31(3):424–35. doi: 10.1016/j.ccell.2017.01.014 28292440 PMC5386178

[55] Yang G, Francis D, Krycer JR, Larance M, Zhang Z, Novotny CJ, et al. Dissecting the biology of mTORC1 beyond rapamycin. Sci Signal. 2021;14(701):eabe0161. doi: 10.1126/scisignal.abe0161 34546793 PMC8580572

[56] Tye BW, Commins N, Ryazanova LV, Wühr M, Springer M, Pincus D, et al. Proteotoxicity from aberrant ribosome biogenesis compromises cell fitness. Elife. 2019;8:e43002. doi: 10.7554/eLife.43002 30843788 PMC6453566

[57] Begum G, Yan HQ, Li L, Singh A, Dixon CE, Sun D. Docosahexaenoic acid reduces ER stress and abnormal protein accumulation and improves neuronal function following traumatic brain injury. J Neurosci. 2014;34(10):3743–55. doi: 10.1523/JNEUROSCI.2872-13.2014 24599472 PMC6608987

[58] Yang Y, Lu D, Wang M, Liu G, Feng Y, Ren Y, et al. Endoplasmic reticulum stress and the unfolded protein response: emerging regulators in progression of traumatic brain injury. Cell Death Dis. 2024;15(2):156. doi: 10.1038/s41419-024-06515-x 38378666 PMC10879178

[59] Ni C, Buszczak M. The homeostatic regulation of ribosome biogenesis. Semin Cell Dev Biol. 2023;136:13–26. doi: 10.1016/j.semcdb.2022.03.043 35440410 PMC9569395

[60] Olatona OA, Sterben SP, Kansakar SBS, Symes AJ, Liaudanskaya V. Mitochondria: the hidden engines of traumatic brain injury-driven neurodegeneration. Front Cell Neurosci. 2025;19:1570596. doi: 10.3389/fncel.2025.1570596 40417416 PMC12098645

[61] Singh LN, Kao S-H, Wallace DC. Unlocking the complexity of mitochondrial DNA: a key to understanding neurodegenerative disease caused by injury. Cells. 2021;10(12):3460. doi: 10.3390/cells10123460 34943968 PMC8715673

[62] Baral SS, Lieux ME, DiMario PJ. Nucleolar stress in *Drosophila* neuroblasts, a model for human ribosomopathies. Biol Open. 2020;9(4):bio046565. doi: 10.1242/bio.046565 32184230 PMC7197718

[63] Lessard F, Igelmann S, Trahan C, Huot G, Saint-Germain E, Mignacca L, et al. Senescence-associated ribosome biogenesis defects contributes to cell cycle arrest through the Rb pathway. Nat Cell Biol. 2018;20(7):789–99. doi: 10.1038/s41556-018-0127-y 29941930

[64] Sirozh O, Saez-Mas A, Jung B, Sanchez-Burgos L, Zarzuela E, Rodrigo-Perez S, et al. Nucleolar stress caused by arginine-rich peptides triggers a ribosomopathy and accelerates aging in mice. Mol Cell. 2024;84(8):1527-1540.e7. doi: 10.1016/j.molcel.2024.02.031 38521064

[65] Blommer J, Fischer MC, Olszewski AR, Katzenberger RJ, Ganetzky B, Wassarman DA. Ketogenic diet reduces early mortality following traumatic brain injury in Drosophila via the PPARγ ortholog Eip75B. PLoS One. 2021;16(10):e0258873. doi: 10.1371/journal.pone.0258873 34699541 PMC8547619

